# Expandable Layered Hybrid Materials Based on Individual 1D Metalorganic Nanoribbons

**DOI:** 10.3390/ma12121953

**Published:** 2019-06-17

**Authors:** Jose Maria Moreno, Alexandra Velty, Urbano Diaz

**Affiliations:** Instituto de Tecnología Química, Universitat Politècnica de València-Consejo Superior de Investigaciones Científicas, Avenida de los Naranjos s/n, E-46022 Valencia, Spain; josemmorenorodriguez@gmail.com (J.M.M.); avelty@itq.upv.es (A.V.)

**Keywords:** MOFs, layered materials, hybrids, monocarboxylate spacers, growing inhibitors, exfoliation

## Abstract

Different metalorganic lamellar hybrid materials based on associated nanoribbons were synthesized by the use of alkyl–benzyl monocarboxylate spacers, containing alkyl tails with variable lengths, which acted like structural growing inhibitors. These molecular agents were perpendicularly located and coordinated to aluminium nodes in the interlayer space, controlling the separation between individual structure sub-units. The hybrid materials were studied by X-ray diffraction (XRD), chemical and thermogravimetrical analysis (TGA), nuclear magnetic resonance (NMR) and infrared spectroscopy (IR), and field emission scanning electron microscopy (FESEM)/transmission electron microscopy (TEM), showing their physicochemical properties. The specific capacity of the metalorganic materials to be exfoliated through post-synthesis treatments, using several solvents due to the presence of 1D structure sub-units and a marked hydrophobic nature, was also evidenced.

## 1. Introduction

Nanometric sheets are essential builders to prepare new families of adjustable layered materials; their synthesis, study and applicability is an accurate difficulty in material science, and one can take advantage of their capability to be altered, replaced and, finally, used as structural part of novel hybrid composite materials [1,2]. Inorganic nanosheets, based on metallic oxides or silica tetrahedra, are fundamental builder units, which conform to several types of layered solids with different long-order morphologies, spatial dispositions and physico-chemical characteristics. Layered metallic oxides (titanates, vanadates, molybdates, etc.), clays (such as montmorillonite), anionic clays (layered double hydroxides, LDHs) or silicoaluminates (such as magadiite or kenyaite) are representative families of layered solids based on the regular and homogeneous ordered distribution of inorganic sheets [3,4,5,6,7].

It is known that the capacity of lamellar materials to be altered by the spatial modification of individual sheets through swelling, intercalation or exfoliation methodologies, in order to generate more accessible materials, and it is also possible to incorporate additional functional sites during these post-synthesis treatments [8,9,10]. The ionic exchange ability of the lamellar materials also facilitates the inclusion of stabilized soluble organocatalysts between the inorganic layers. Inside this field, important advances have been carried out, starting from layered zeolitic precursors (preferably with MWW, FER or NSI topologies, following IZA codes) [11,12]. In this case, delaminated zeolites were obtained, after swelling and sonication processes, combining the accessibility of mesoporous materials with the high stability and reactivity of conventional zeolites. The possibility to covalently anchor specific organometallic complexes, enzymes or chiral pending organic moieties onto the surface of zeolitic layers favoured the formation of accessible layered hybrid materials with catalytic applications in fine chemistry or petrochemistry. Additionally, the introduction of specific functions in organic linkers, located between ordered zeolitic sheets, during the pillarization process, combined with the intrinsic active centers present in the inorganic layers, facilitated the preparation of bi-functional acid-base layered hybrid materials capable of performing a one-pot two-step cascade or consecutive catalytic reactions, such as the hydrolysis of hemiacetals followed by C-C bond forming reactions [13].

In the last years, important advances were carried out in the synthesis methodologies to generate lamellar hybrid materials in only one step, avoiding long-expensive processes where the synthesis of a layered precursor, ionic exchange, swelling, pillarization, delamination, or exfoliation and extraction steps are necessary to obtain the final catalysts with different active functions included into the framework. In this sense, layered hybrid aluminosilicates, named ECS-type materials, based on organic pillars and inorganic sheets formed by [AlO_4_] and [SiO_3_C] structural units, were directly synthesized in a one-step process with low Si/Al molar ratios (close to 1), strong alkaline media and under hydrothermal synthesis conditions, using bridged silsesquioxanes (disilanes) as organosilicon sources [13,14].

Following a similar tendency to directly obtain new lamellar solids, novel families of layered zeolitic materials (preferably with an MFI and MWW topology) were prepared, in only a one-step process, by using specific and dual organic structural directing agents (SDAs) during the hydrothermal synthesis of conventional zeolites. Specifically, in this case, organic compounds formed by long hydrocarbon tails and polar alkylammonium heads are used as templates favouring the synthesis of small sheet-type crystallites of zeolites, which are orderly separated. The high specificity of SDAs used during the hydrothermal synthesis favoured the crystallization of zeolitic small crystals that are organized as ordered layers due to the dual function of the organic templates as structural directing agents (charged head) and growing molecular 3D inhibitors (long hydrocarbonated tail) [10].

Specifically, in MOF-type metalorganic structures, it is not too habitual to obtain the formation of modifiable layered precursors based on ordered individual nanosheets, spatially organized by electrostatic interactions, these being normally obtained 3D architectures formed by the coordination linkage established between metallic nodes and rigid bi-carboxylate organic spacers [15,16,17]. However, different bi-dimensional layered-pillared metalorganic frameworks can directly be synthesized due to the generation of organic-inorganic layers, based on isophthalic acid units interacting with metallic nodes, which are functionalized with rigid pillar ligands, such as pyridyl- or triazolyl-types with N-donor moieties [18,19,20]. In this case, this type of 2D rigid frameworks are unmodifiable from a structural point of view through post-synthesis treatments, exhibiting several pore volumes and pore sizes depending on the length of the pillar ligands, resulting in gas adsorption capacities.

On the other hand, as far as we know, the synthesis of expandable lamellar metalorganic precursors based on individual organic-inorganic layers, both by hydrothermal or solvothermal methodologies and by using modified organic linkers as growing inhibitors, is not habitual in the literature. Only preliminary works that are related to the generation of MOF nanosheets via the control of growth kinetics during the synthesis process have been described [21]. Despite this, the existence of 3D conventional MOFs, which exhibit a layered structural morphology taking into account the spatial distribution of their structure building units, would confirm that it is possible to prepare metalorganic structures with lamellar frameworks. Particularly, from a morphological point of view, these metalorganic structures are based on infinite 1D inorganic M-O-M chains, based on individual metal nodes, separated by aryl–dicarboxylate linkers perpendicularly located to metallic cluster arrangements. This morphology is observed in several well-known MOFs such as MIL-53(Al), DUT-4, DUT-5, DUT-8, MOF-Zn-DABCO, Cu-(tpa) or MIL-68(Al), among others, and it is possible to detect low-dimensional inorganic structure sub-domains in their architecture [17,22,23,24,25,26].

Taking account the above information and considering the high hydrothermal stability of MIL-53(Al), we have, in the present work, synthesized several lamellar organic-inorganic solids in only a one-step process, formed by 1D ordered individual aluminium cluster-type nanoribbons separated by specific alkyl arylic monocarboxylate units, which play the role of molecular spacers, perpendicularly located to inorganic clusters. These organic spacers present hydrocarbonated tails with variable lengths, and it is possible to control the separation between the different inorganic low dimensional structure units, inhibiting the 3D growth of conventional MIL-53(Al)-type MOFs. Post-synthesis processes with several solvents permitted the complete expansion of starting lamellar metalorganic structures, rendering possible the formation of exfoliated hybrid compounds formed by individual disordered metalorganic 1D nanostructures.

## 2. Materials and Methods

### 2.1. Synthesis of MIL-53 (Al), Al-ITQ-EB, Al-ITQ-HB and Al-ITQ-DB Metalorganic Materials

The synthesis methodology of this family of materials was carried out by mixing 3.1 mmol of organic linkers: 1,4–bencenedicarboxylic acid (BDC, Aldrich), 4–ethylbenzoic acid (EB, Aldrich), 4–heptylbenzoic acid (HB, Aldrich) or 4–dodecylbenzoic acid (DB, Alfa Aesar), and aluminium reagent (AlCl_3_.6H_2_O, 3.1 mmol) in 15 mL of DMF (Dimethylformamide, Acros Organics), each one separately. When both solutions were totally homogeneous, they were mixed together. Then, the formed solution was charged into a stainless autoclave and heated at 150 °C for 24 h under autogeneous pressure and static conditions. After this, the autoclave was cooled to room temperature and the obtained solid was isolated by filtration. Particularly, hybrid material based on BDC was filtrated with DMF and washed with hot DMF at 150 °C by reflux. Finally, the material was washed with methanol to remove the excess DMF and dried at 100 °C. However, hybrid materials that were formed by monocarboxylate organic spacers were separated by filtration with distilled water and washed several times with methanol, before being dried at room temperature.

### 2.2. Characterization Techniques

An XRD analysis was performed with a Philips X’PERT diffractometer (Panalytical) equipped with a detector and a secondary graphite monochromator. The data were collected stepwise over the 2° ≤ 2θ ≤ 20° angular region, with steps of 0.02° 2θ, 20 s/step accumulation time and CuKα (λ = 1.54178 Å) radiation. Transmission electron microscopy (TEM) micrographs were acquired with a JEM2100F electron microscope (JEOL Ltd., Tokyo, Japan) operating at 200 keV. The samples were treated through the dispersion of the powders onto carbon copper grids. The C, N and H contents were estimated with a model 1106 elemental analyzer (Carlo Erba), while the Al contents were determined through atomic absorption spectroscopy using Spectra AA 10 Plus (Varian). Thermogravimetric and differential thermal analyses (TGA-DTA) were carried out in an air stream with a TGA/SDTA 851E analyzer (Metler Toledo). An Ar adsorption isotherm was performed at –186 °C in an ASAP 2010 apparatus (Micromeritics), and the samples were previously treated under vacuum at 100 °C overnight. The Hörvath-Kawazoe equation as used to analyze the pore size distribution. IR spectra were collected in a Nicolet 710 spectrometer (Thermo Scientific) (4 cm^−1^ resolution), employing a conventional greaseless cell. Wafers of 10 mg·cm^−2^ were outgassed at 100 °C overnight. Solid state MAS-NMR spectra were obtained at room temperature in a AV-400 III HD spectrometer (Bruker, Billerica, MA, USA). ^27^Al spectra were obtained using a 4 mm BL-4 probe (Bruker). Pulses of 0.5 μs to flip the magnetization π/20 rad, as well as a recycle delay of 1 s were employed. The ^1^H to ^13^C cross-polarization (CP) spectra were recorded by using a 90° pulse for 1H of 5 μs, a contact time of 2 ms, and a recycle of 3 s. The ^13^C spectra were recorded with a 4 mm Bruker BL-4 probe and at a sample spinning rate of 10 kHz. The ^1^H to ^27^Al cross-polarization (CP) spectra were collected with a bruker 3.2 mm probe employing an rf-field for ^27^Al of 8 kHz, spinning the sample at 16 kHz, a contact time of 1 ms, and a recycle of 3 s. ^13^C and ^27^Al were referred to adamantane and an aqueous solution of Al(NO_3_)_3_, respectively.

## 3. Results and Discussion

**Scheme 1:**
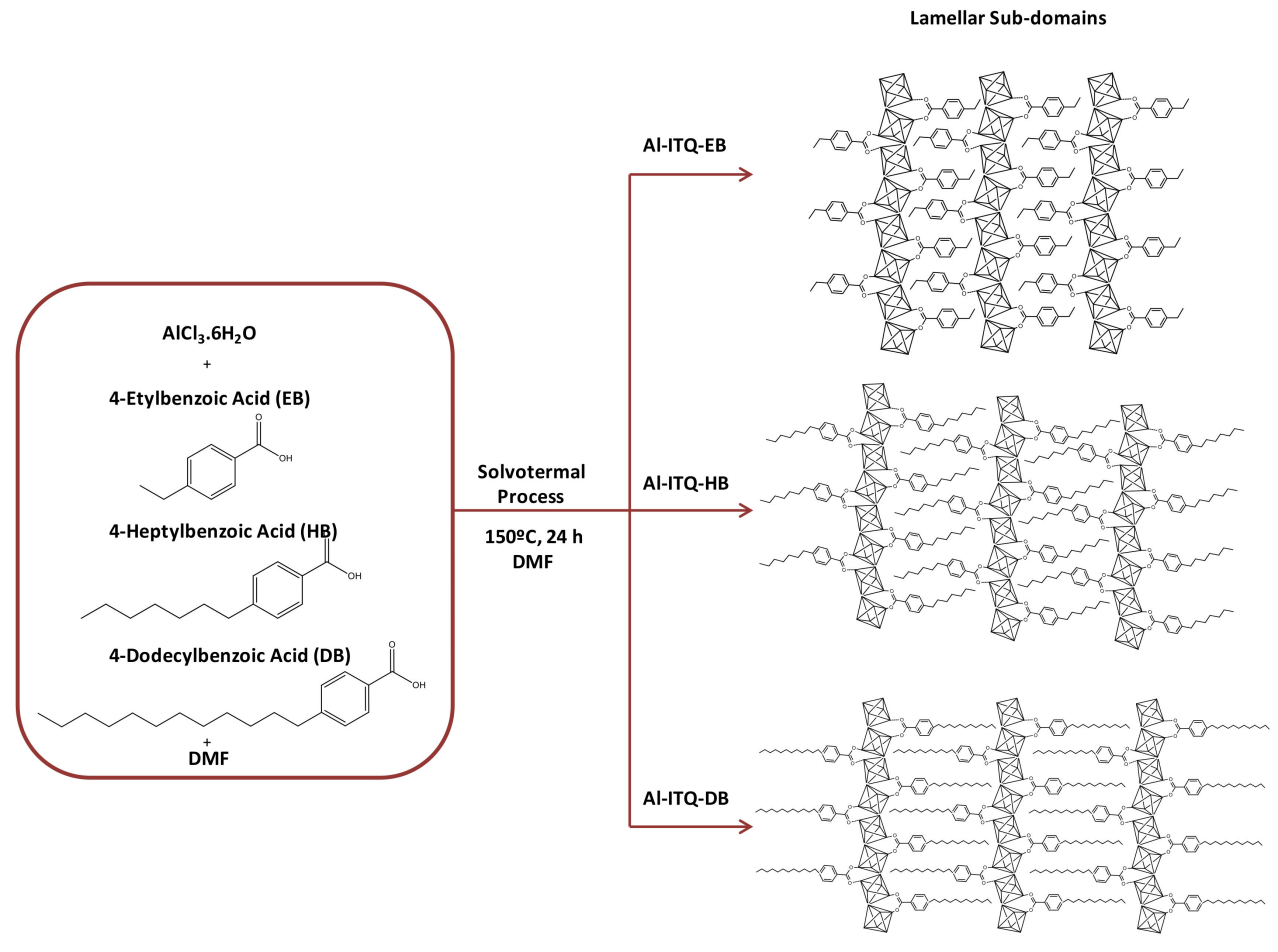
Synthesis route to synthesize different hybrid Al-MOFs materials formed by 1D structural sub-domains.

The procedure that was followed to prepare the layered hybrid materials formed by ordered individual and expandable low dimensional metalorganic sub-units was based on the use of specific organic spacers that contain only one attaching terminal group that interacted with inorganic metallic clusters by stable coordination bonds. In this study, different alkyl benzene monocarboxylate molecules with hydrocarbonated tails of variable lengths (ethyl, heptyl and dodecyl), located in the para position, were employed as organic spacers, being named EB, HB and DB (see Scheme 1). These building spacers were used in the place of more standard rigid aryl-type dicarboxylate linkers, normally employed in the synthesis of conventional 3D MOF architectures. The solvothermal conditions, together with aluminium chloride, dimethylformamide (DMF) and EB, HB or DB as the selected reagents in the synthesis gel, facilitated the generation of ordered lamellar hybrid materials probably formed by metalorganic layers separated by the alkyl benzene monocarboxylate spacers, which are perpendicularly placed to the inorganic clusters. The post-synthesis step of putting together lamellar architectures with non-polar solvents (such as dichloromethane or chloroform) facilitated the expansion, delamination and effective exfoliation of single metalorganic 1D nanoribbons, which were forming the pristine lamellar materials.

**Scheme 2:**
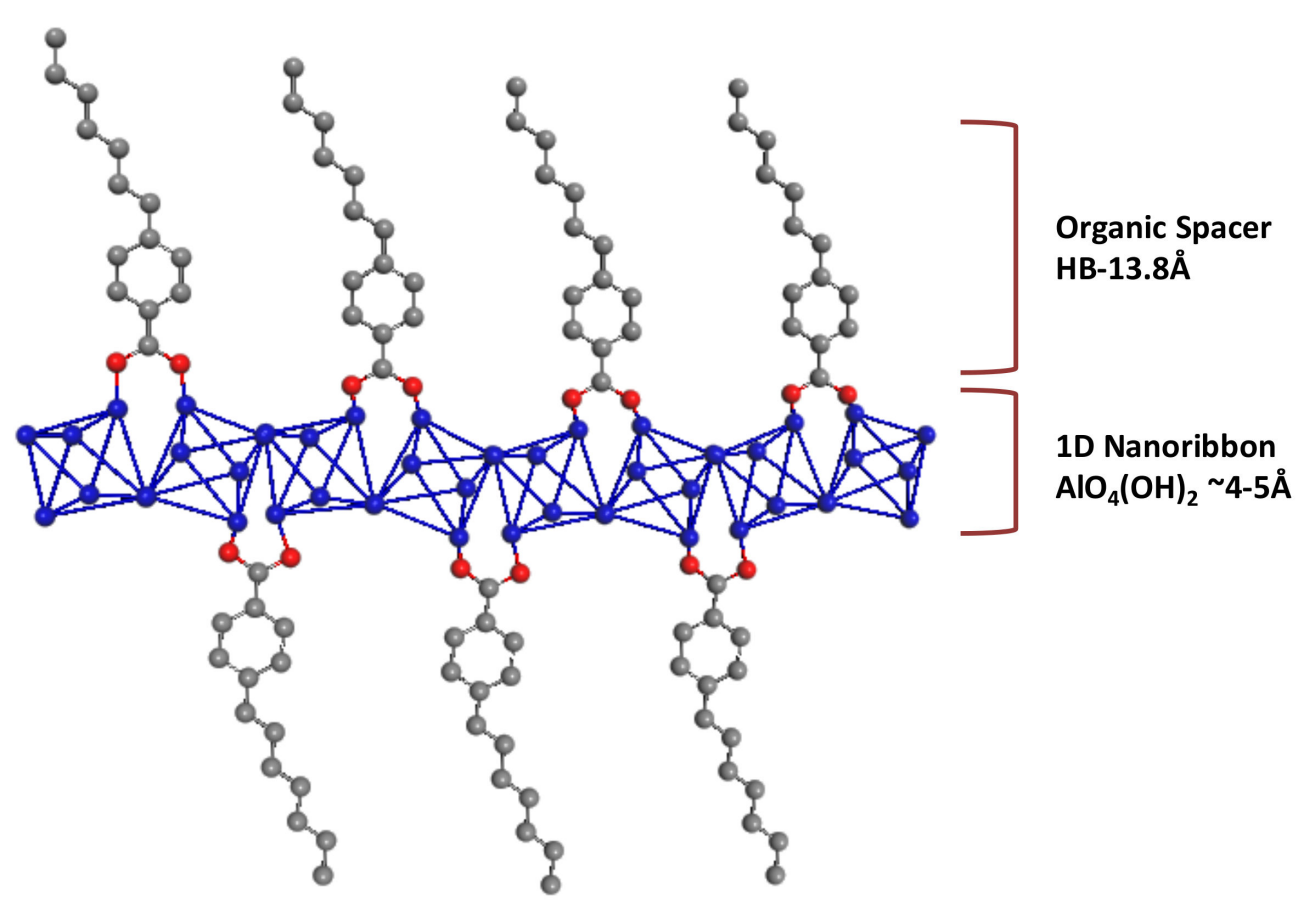
Schematic representation of the hybrid structuration of Al-ITQ-HB material based on the layers formed by the association of 1D nanoribbons, which contain alkyl–benzyl monocarboxylate spacers (HB), perpendicularly located to AlO_4_(OH)_2_ units.

The followed synthesis route to prepare this novel type of hybrid lamellar materials is shown in Scheme 1, together with the representation of individual organic-inorganic nanoribbons, highlighting the different basal spaces detected between the layers depending on the organic spacer length used during the solvothermal process, which would play the role of effective growing inhibitors of the more conventional 3D metalorganic architectures. As observed in this figure drawing, theoretically, the individual layers would be based on consecutive corner-sharing octahedral AlO_4_(OH)_2_ sub-domains, forming a 1D inorganic chain distanced by alkyl benzene monocarboxylate ligands, which surround the metallic nodes (Scheme 2). Therefore, the coordinative interaction established between inorganic chains and organic spacers around the metallic nodes probably favoured the formation of each individual metalorganic sheet, which would be the basis for this type of hybrid lamellar material. Specifically, in these structural sub-domains, octahedral building units would be connected together through two hydroxyl groups placed in *trans* positions, the rest of the positions being coordinatively bound to the alkyl-substituted benzoate spacers, shared with adjacent octahedra through a Kagomé structural conformation, typical of trivalent metallic clusters [27,28]. This topology, based on 1D sub-domains, facilitated the preparation of derived 2D MOF-type structures with the molecules of monocarboxylate organic spacers perpendicularly linked to each metallic cluster (Scheme 2).

**Figure 1:**
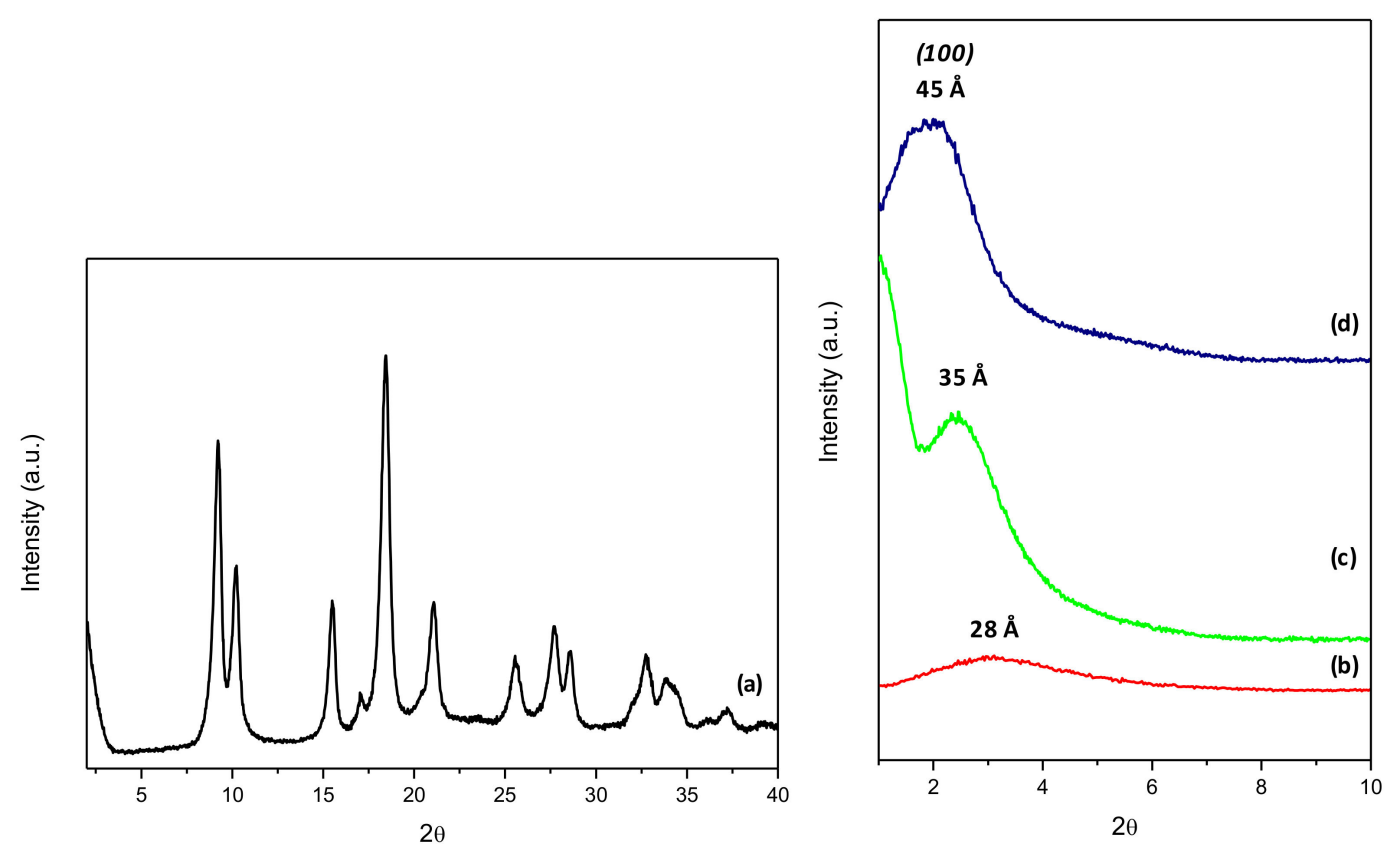
The X-ray diffraction (XRD) patterns of hybrid materials: (**a**) MIL-53(Al), (**b**) Al-ITQ-EB, (**c**) Al-ITQ-HB and (**d**) Al-ITQ-DB.

The XRD patterns of the organic-inorganic materials prepared in the presence of aluminium salts and *para*-alkyl arylic spacers showed that a layered organization was achieved, given that a (*100*) low angle diffraction band was detectable, characteristic of lamellar materials based on individual sheets perpendicularly located to the *a* axis (Figure 1). In all cases, the existence of this reflection would confirm that a certain regularity was achieved in the molecular distance (basal space) observed between the consecutively ordered organic-inorganic sheets (Scheme 2). Specifically, the layered materials prepared with ethyl– (EB), heptyl– (HB) and dodecyl– (DB) benzene monocarboxylic acids as organic spacers (so-called Al-ITQ-EB, Al-ITQ-HB and Al-ITQ-DB) showed one evident low angle (*100*) diffraction band centred at ~3.0, ~2.5 and ~2.0 2θ degrees, corresponding to molecular basal spaces of ~28.0 Å, ~35.0 Å and ~45.0 Å, respectively. Taking account the fact that the thickness of each single 1D aluminium-chain is near to 4–5 Å and that the organic spacers (EB, HB and DB), perpendicularly located with respect to the inorganic AlO_4_(OH)_2_ nanoribbons exhibited molecular lengths of 6.3 Å, 13.8 Å and 20.0 Å, respectively, overlapping phenomena between the organic moieties present in the interlayer space could be happening, probably due to the presence of non-coordinated alkyl–benzoate spacers located between the individual layers (Scheme 1). Alternatively, a coordinative association of several sheets, through the stable interaction of corner sharing hydroxyl groups present in [AlO_6_] octahedra, as a means of forming inorganic sub-domains with a greater thickness, could also be considered. Furthermore, in comparison with the XRD pattern of the standard 3D MIL-53(Al) MOF-type structure (Figure 1), the lamellar materials exhibited a completely different diffractogram with a only characteristic low angle *(100)* band connected with the disappearance of typical (*hkl*) MIL-53(Al) diffraction bands, which indicated the complete absence of a 3D structuration and the generation of low dimensional metalorganic sub-structures. However, the XRD diffractograms of the Al-ITQ-EB, Al-ITQ-HB and Al-ITQ-DB lamellar hybrid materials did not show high angle diffraction bands, due to (*0kl*) reflections associated to the *bc* plane of each individual layer, indicating that the structure sub-domains exhibited a relative low crystallinity.

Additional experiments were also performed using the microwave technique; the synthesis time to obtain hybrid materials based on 1D nanoribbons was reduced from 24 h to only 15 min. This result evidenced the effectiveness of the microwave system to reduce the period of nucleation and crystallization steps during the synthesis processes without modifying the morphological and physico-chemical properties of the solids (see Figure S1 in Supplementary Materials).

**Figure 2:**
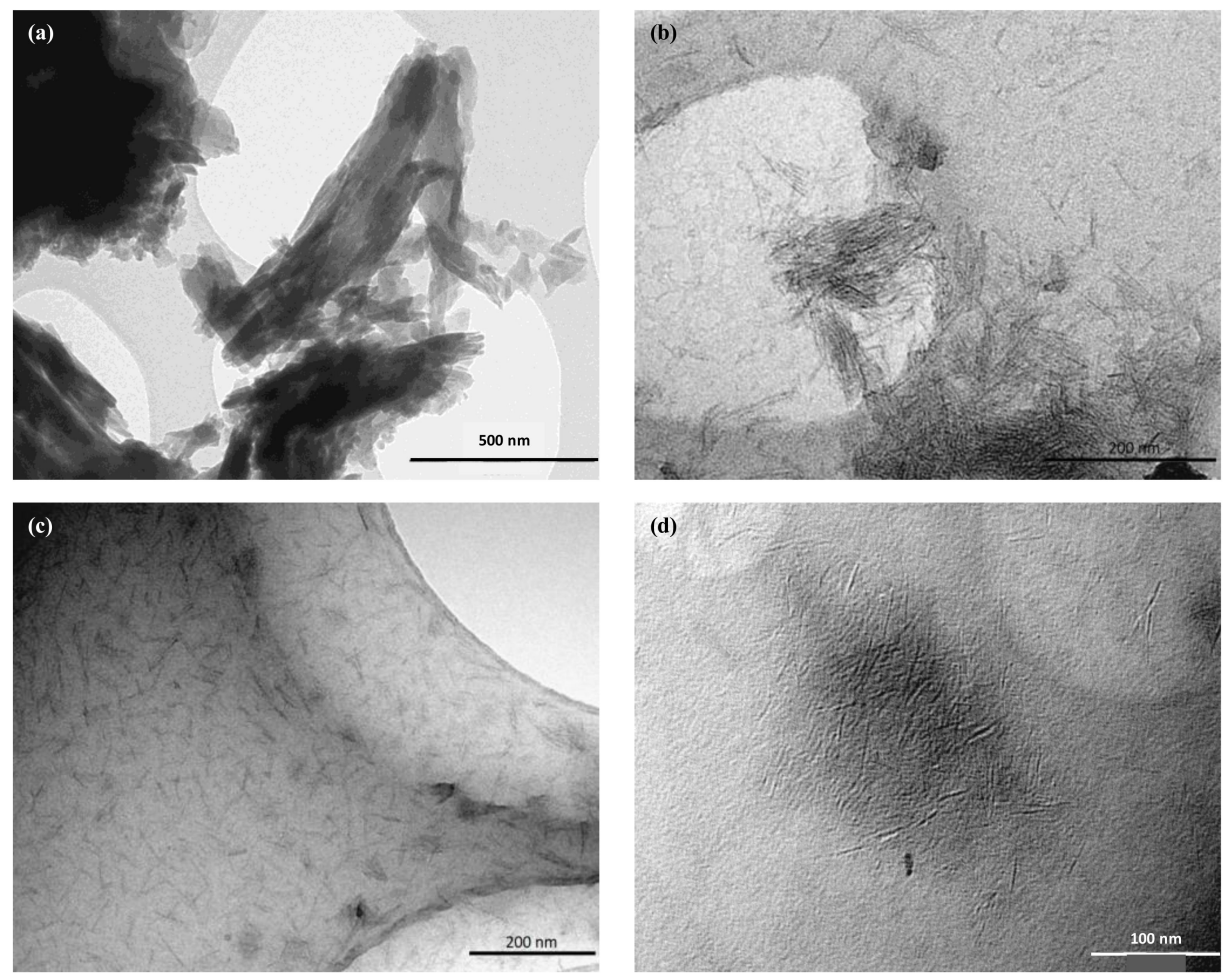
High resolution transmission electron microscopy (HRTEM) images: (**a**) MIL-53(Al), (**b**) Al-ITQ-EB, (**c**) Al-ITQ-HB, and (**d**) Al-ITQ-DB samples treated in dichloromethane. Scale bars correspond to 500 nm for (**a**), 200 nm for (**b**) and (**c**), and 100 nm for (**d**) micrographs.

The lamellar morphology of the sub-domains of Al-ITQ-EB, Al-ITQ-HB and Al-ITQ-DB materials was confirmed by HRTEM micrographs (Figure 2 and Figure S2 in the Supplementary Materials); the presence of individual nanoribbons expanded into the organic solvent was detected. Remarkable differences were observed between the hybrid materials synthesized here with monocarboxylate organic spacers and the standard 3D MIL-53(Al) MOF because the latter was based on dense elongated prism-like crystallites; in this type of hybrid structures, one could observe the low dimensional character associated with a homogenous association of individual aluminium nanoribbons present in their structure.

Interestingly, the as-synthesized layered hybrid materials were easily dispersed in polar organic solvents (Figure 2), such as dichloromethane or chloroform; stable and durable suspensions were generated, where disordered individual 1D nanoribbons formed by [AlO_6_] octahedra were detectable with approximately 4–5 Å of thickness, corresponding to the inorganic counterpart of each individual metalorganic structure sub-unit (Figure 2). This behaviour confirmed the expandable ability of this lamellar metalorganic material; it is possible to completely separate each single sheet and the associated 1D structural sub-domains through the exfoliation of the starting ordered hybrid materials (Al-ITQ-EB, Al-ITQ-HB and Al-ITQ-DB) in the presence of polar organic solvents. During this exfoliation process, a partial coordination exchange between alkyl–benzyl monocarboxylate ligands and organic solvent molecules could occur, as normally happens in standard 3D metalorganic materials [29,30,31]. A delamination process was favoured by the marked hydrophobic nature of the layered hybrid materials initially prepared, associated to the elevated amount of long hydrocarbonated tails located around each aluminium-clusters chain. However, it was observed that the exfoliation level that was achieved was more marked when the organic spacer used during the synthesis process was longer, i.e., with heptyl- or dodecyl- tails. In these cases, the electrostatic interaction between the individual metalorganic nanosheets was probably minimized, facilitating the exfoliation effect.

From the elemental CHNS analysis shown in Table 1, we calculated the amount of organic content included in the hybrid metalorganic materials coordinated in each individual 1D nanoribbon from the different organic spacers employed in the solvothermal synthesis procedure. The results indicated that the organic counterpart contribution oscillated between 20 % wt and 50 % wt for the organic-inorganic solids, being higher when the ligands contained longer hydrocarbonated tails, such as HB and DB spacers. The organic content of the layered materials (HB and DB) was comparable to the standard 3D MIL-53(Al) MOF (~45 % wt) because the organic linkers were shared between two aluminium clusters in the three-dimensional solid, while practically the same amount of organic ligands was necessary in the Al-ITQ-HB and Al-ITQ-DB hybrid solids to form individual 1D sub-domains (Scheme 1). This fact would be indicative of the presence of one or two opposite coordinated monocarboxylate organic spacers on both sides of the 1D Al-chains, being shared with contiguous aluminium octahedra (Scheme 2), such as occurs in the conventional 3D MIL-53(Al) MOF-type materials. The variation of the organic content in the different hybrid materials favoured that the C/Al molar ratio was not the same in all cases. Furthermore, the non-presence of nitrogen in the samples confirmed that all dimethylformamide (DMF), which was employed as solvent in the preparation processes, was eliminated in the last washing step and was not present in the final layered metalorganic materials.

**Figure 3:**
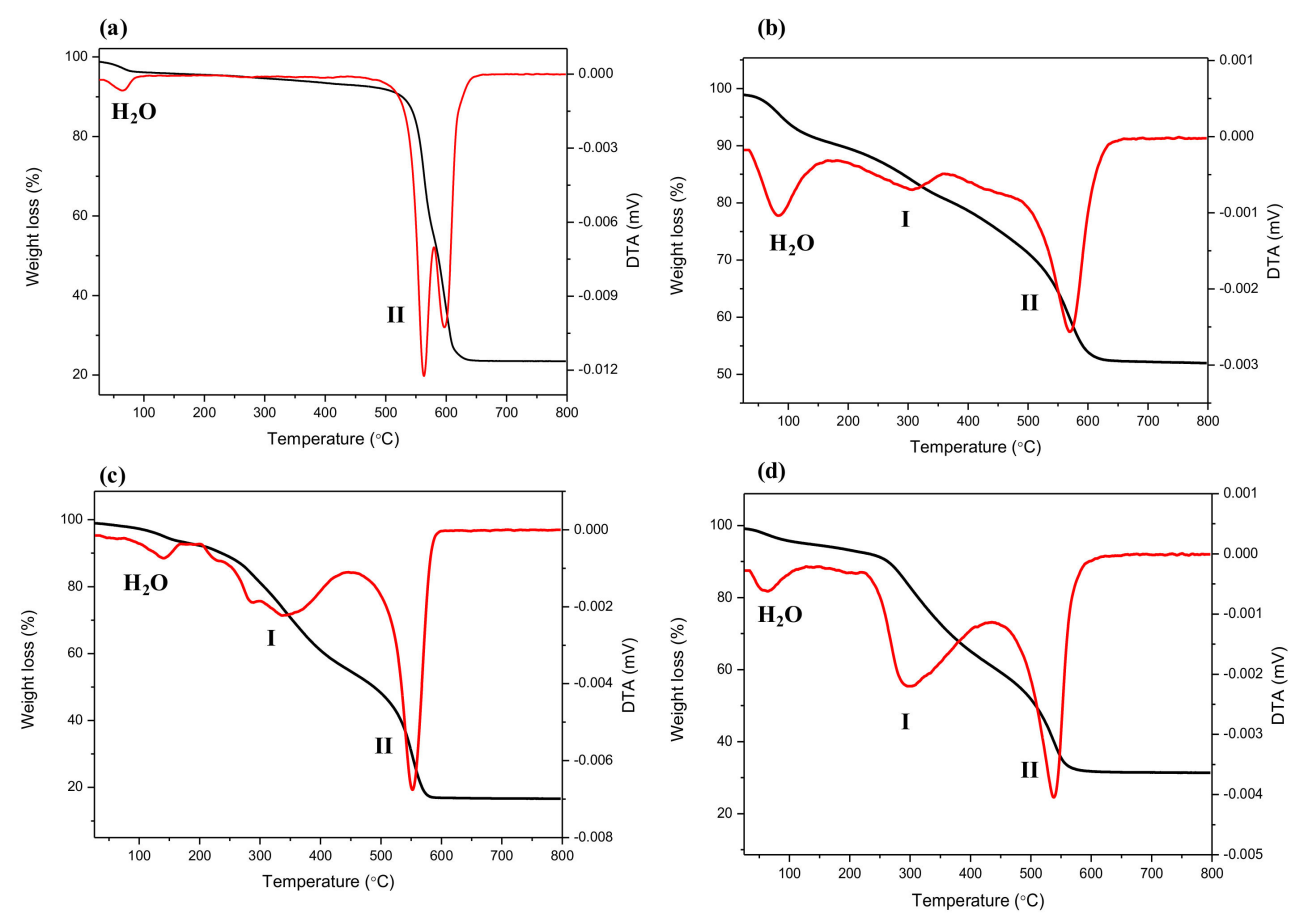
Thermogravimetric (TG) and derivative (DTA) curves: (**a**) MIL-53(Al), (**b**) Al-ITQ-EB, (**c**) Al-ITQ-HB and (**d**) Al-ITQ-DB.

Figure 3 shows the weight losses and the different derivatives (TGAs and DTA curves) with the temperature for the obtained low dimensional hybrid materials. These results allowed for the establishing, not only of the content of the organic spacers introduced in the Al-ITQ-EB, Al-ITQ-HB and Al-ITQ-DB solids, but also of the hydrothermal stability of the organic-inorganic structures. In all of the hybrid solids, without considering the contribution of hydration water and DMF used as solvent in the synthesis processes (both detected at around 80–150 °C), it was possible to detect a most important weight loss (II), close to 550 °C, due to the existence of arylic units from *para*-alkyl benzene carboxylate molecules employed as alkyl–benzoate spacers; this weight loss was also observed for standard 3D MIL-53(Al) MOF due to the benzene dicarboxylate (BDC) linkers present in the network. From the DTA curves (Figure 3), it was also possible to observe the gradual thermal decomposition of hydrocarbon tails (ethyl-, heptyl-, dodecyl-), present in the respective organic spacers, associated with the weight loss detected between 250 °C and 400 °C (I), and this was more appreciable when the alkyl tail was longer (Al-ITQ-DB). This latter weight loss could also be associated to the presence of non-coordinated organic spacers. It should be mentioned that the organic content estimated from the thermogravimetric curves, similarly to the results collected from the CHNS elemental analysis (see Table 1), confirmed the presence of several organic ligands in the hybrid materials. However, the increase in organic content detected from TGA could be associated with dehydroxylation water produced at high temperatures (650 °C), resulting from the thermal decomposition of aluminium (oxy)hydroxide nodes.

**Figure 4:**
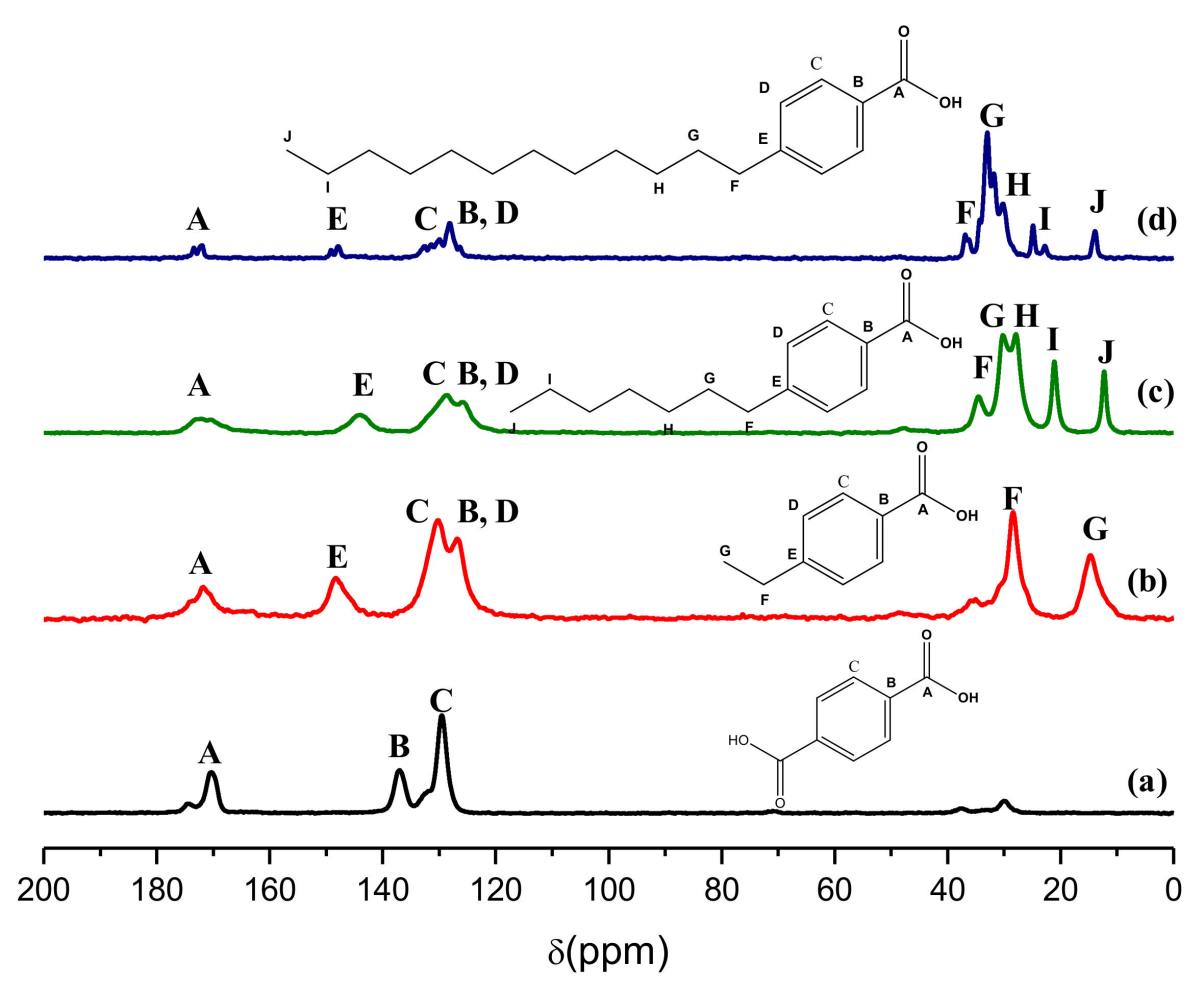
^13^C Magic Angle Spinning (MAS) Nuclear Magnetic Resonance (NMR) spectra: (**a**) MIL-53(Al), (**b**) Al-ITQ-EB, (**c**) Al-ITQ-HB and (**d**) Al-ITQ-DB.

The ^13^C CP/MAS NMR spectra of the metalorganic hybrid organic-inorganic solids are shown in Figure 4. From the obtained results, we corroborated the complete integrity of the organic spacers (EB, HB and DB) after the applied solvothermal synthesis procedures, since all of the carbon atoms, including the hydrocarbon tails, directly bonded to the benzene monocarboxylate units and were perfectly assigned in the ^13^C NMR spectra (see the different insets in Figure 4). Specifically, in the hybrid materials and conventional MIL-53(Al) MOF, chemical shifts of around 130 and 170 ppm were detected, associated with the carbon atoms of benzene rings and attached benzyl-carboxylate functions, respectively. In the case of the hybrid solids based on the 1D sub-domains (Al-ITQ-EB, Al-ITQ-HB and Al-ITQ-DB), different chemical shifts were detected between 10 and 40 ppm, associated with the carbon atoms from the alkyl tails of the respective arylic monocarboxylate spacers, independently of the alkyl chains present in each organic linker. The detected splitting of some chemical shifts could be due to the presence of organic spacers uncoordinated to metallic nodes. Despite this fact, these spectroscopic results corroborated that the organic ligands used during the synthesis process coordinatively attached to aluminium clusters, remaining intact as in the initial chemical conformation, and being finally included in the hybrid solids.

**Figure 5:**
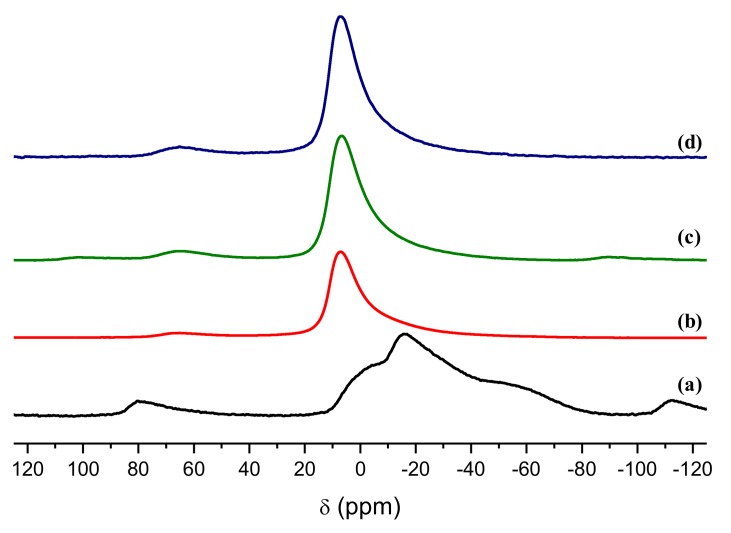
^27^Al MAS NMR spectra: (**a**) MIL-53 (Al), (**b**) Al-ITQ-EB, (**c**) Al-ITQ-HB and (**d**) Al-ITQ-DB.

Furthermore, the ^27^Al MAS NMR of the samples were analysed to characterize with more details the chemical properties of the low dimensional organic-inorganic solids (Figure 5). For conventional 3D MIL-53(Al) MOF, the spectrum showed a characteristic broad quadruple band in the range of –80–0 ppm, previously described in the bibliography for this type of MOFs [17]. Interestingly, in the case of hybrid solids obtained with the EB, HB and DB spacers, the ^27^Al NMR spectra presented a single and well-defined chemical shift at 6.5 ppm, and this signal is close to being typical of the aluminium (oxo)hydroxide sub-units (AlO_4_(OH)_2_) with a regular octahedral environment that corroborated the inorganic counterpart composition of each 1D individual nanoribbon (Scheme 1) [32].

**Figure 6:**
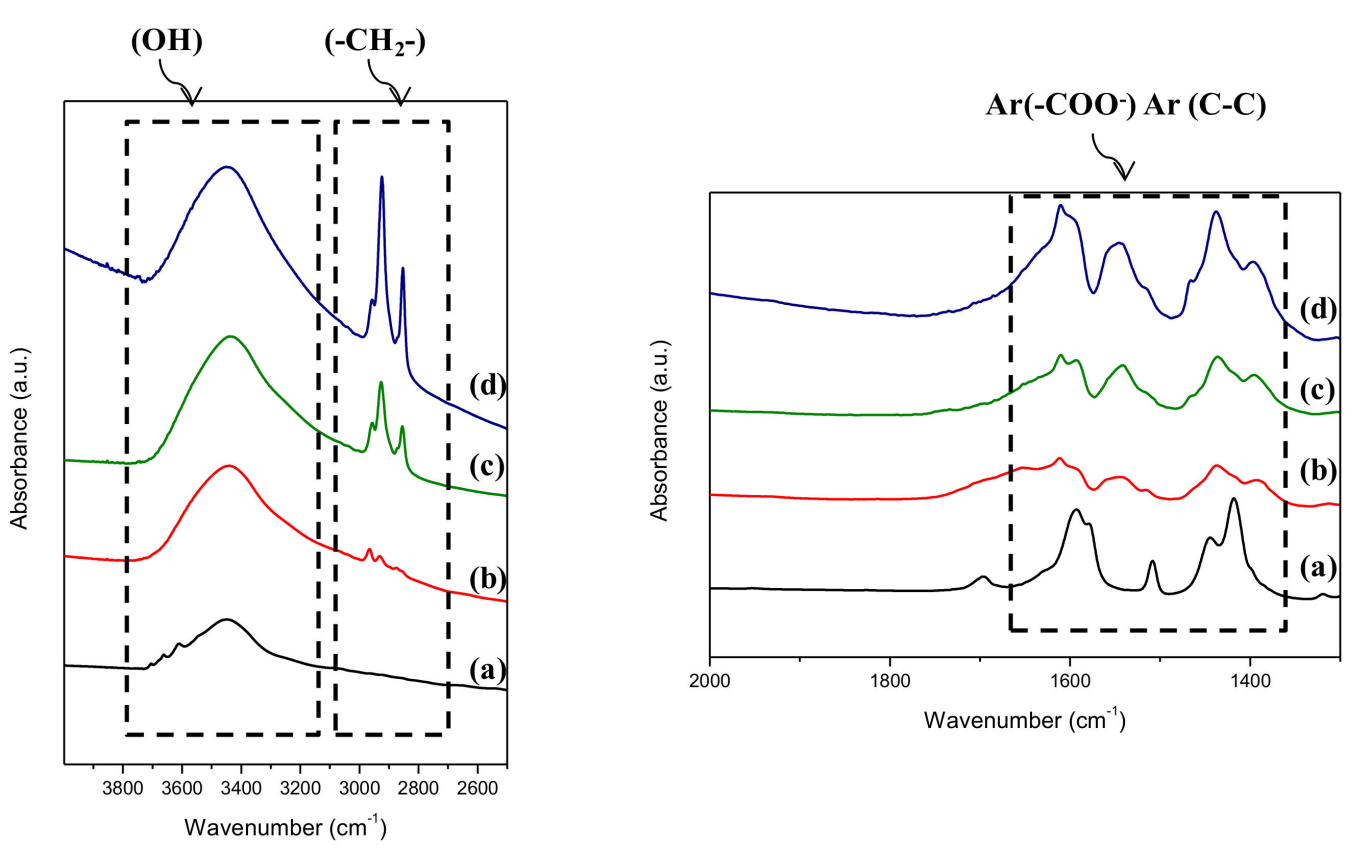
Infrared (IR) spectra: (**a**) MIL-53(Al), (**b**) Al-ITQ-EB, (**c**) Al-ITQ-HB and (**d**) Al-ITQ-DB.

The integrity of the alkyl–benzyl monocarboxylate building units included in the architecture of the layered hybrid MOF-type materials was also confirmed by IR spectroscopy (Figure 6). The presence of aliphatic (C–H) chains from the tails of the organic spacers (EB, HB and DB) was corroborated by different vibrational signals around 3000 cm^−1^ and, specifically, three bands were identified at 2983, 2953, and 2885 cm^−1^, assigned to –CH_2_– groups. In addition, the IR results showed the presence of stretching vibrational signals at 3600–3200 cm^−1^, which corresponded to hydroxyl groups (–OH) associated to hydration phenomena and aluminium oxyhydroxide structural nodes (AlO_4_(OH)_2_). Moreover, a vibrational band assigned to the carboxylate groups of the organic alkyl-substituted benzoate spacers coordinated to Al octahedral was detected at 1400–1600 cm^−1^, characteristic of bidentate chelating ligands. Vibrational bands between 1400 and 1600 cm^−1^ were also assigned to asymmetric stretching vibrations due to benzylic groups present in the organic–inorganic framework [33]. Therefore, the spectroscopic results obtained from the IR and NMR techniques confirmed the presence and integrity of the organic aryl–benzoate spacers conforming to the hybrid architecture of metalorganic MOF-type hybrid materials.

**Figure 7:**
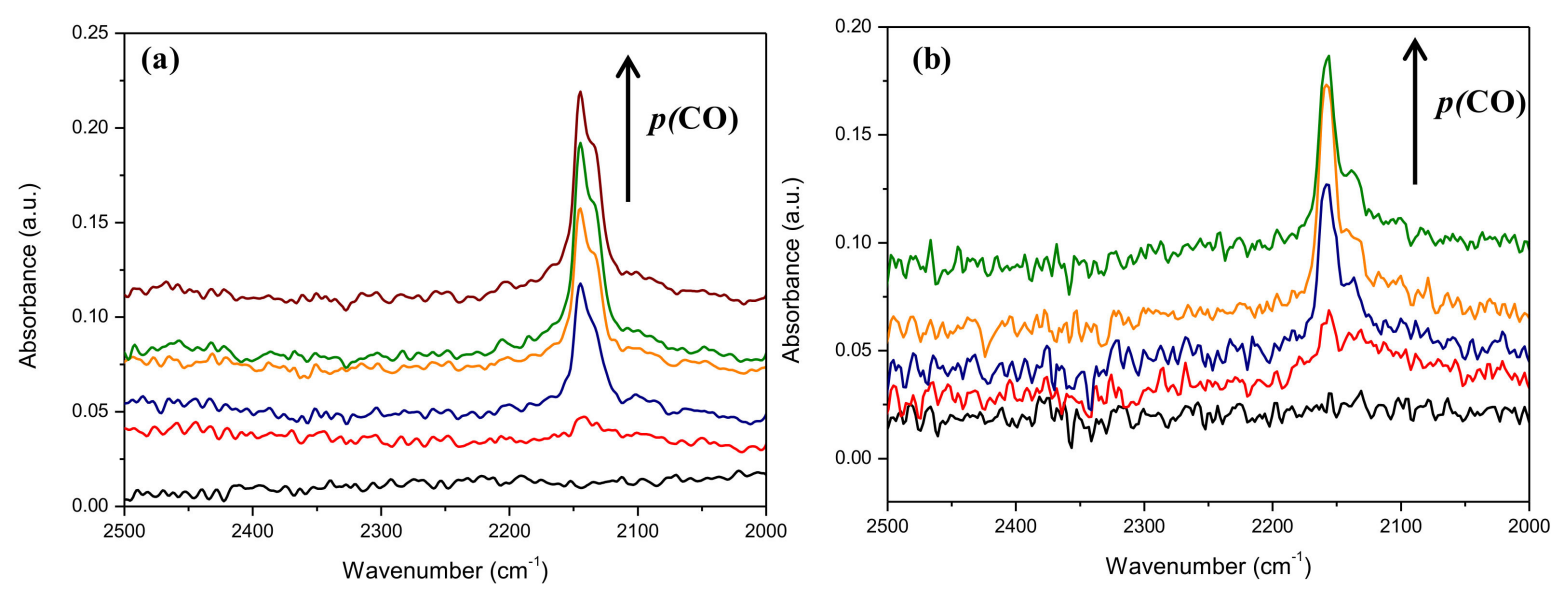
Different Fourier-transform infrared spectroscopy (FTIR) spectra of increasing amounts of CO adsorbed at low temperature (100 K): (**a**) MIL-53 (Al) and (**b**) Al-ITQ-HB (self-supporting wafer).

The Lewis acidity of the derived metalorganic materials was determined from FTIR spectroscopy through CO adsorption. In this way, the adsorption of CO at a low temperature (100 K) was measured by a FTIR technique to obtain data about the Lewis acid centres present in the hybrid organic-inorganic solids (Figure 7). In the MIL-53(Al) and Al-ITQ-HB materials, an absorption band was observed around 2150 cm^−1^, evidencing that weak Lewis acid centres were included into the architecture of the hybrid materials. This fact opens the possibilities that these low-dimensional metalorganic materials could be used as active acid solid catalysts.

The textural properties of the as-synthesized low-dimensional Al-MOF-type hybrid materials were estimated through argon adsorption isotherms (see Figure S3 in the Supplementary Materials). Specifically, in the hybrid organic-inorganic solids obtained together with aryl–monocarboxylate spacers (Al-ITQ-EB, Al-ITQ-HB, Al-ITQ-DB), specific surface areas were reduced (S_BET_ = ~20–30 m^2^·g^−1^), probably due to the poor crystallinity and short-order structuration that were finally achieved. Additionally, the high content of organic spacers placed between single 1D nanoribbons, favouring the blocking of the internal free volume, together with the high hydrophobic nature of the framework, could hinder the optimal adsorption of the gas between the organic–inorganic structure sub-units.

## 4. Conclusions

Several families of lamellar metalorganic (Al-MOF-type) hybrid organic-inorganic solids, formed by associated 1D aluminium nanoribbons, were synthesized through the use of alkyl–benzyl monocarboxylate spacer molecules, containing alkyl tails with variable lengths, which acted as structural growth inhibitor agents of conventional 3D architectures. 1D inorganic nanoribbons of associated aluminium octahedra, coordinated with alkyl-substituted benzoate spacers, would compose each individual organic-inorganic sheet. These structural growing inhibitors were probably perpendicularly located and coordinated to aluminium nodes in the interlayer space, controlling the separation between individual sub-domains. The properties and characteristics of these low dimensional hybrid materials, which exhibited a short-order organization, were confirmed by XRD, chemical analysis, TGA, NMR and IR spectroscopy, and FESEM/TEM microscopy. Interestingly, due to their structure characteristics and marked hydrophobic nature, the specific capacity of the metalorganic materials to expand through post-synthetic processes using several solvents was also observed, and it was possible to generate individual components with a low dimensionality, which could be used to obtain novel films or nanocomposites with catalytic or nanotechnological applications.

## Figures and Tables

**Table 1:** Chemical analysis of the MIL-53(Al), Al-ITQ-EB, Al-ITQ-HB and Al-ITQ-DB materials.

Samples				Cont.Org. ^a^		
	C ^a^	H ^a^	N ^a^	CHN ^b^	ΔTG ^c^	Al ^a^
MIL-53(Al)	43.5	2.4	0	45.9	70.2	13.8
Al-ITQ-EB	21.9	3.9	0	25.8	38.1	27.8
Al-ITQ-HB	34.4	5.6	0.3	40.3	54.1	11.1
Al-ITQ-DB	44.3	7.4	0	51.7	61.0	17.3

^a^ Percentage in weight (% wt); ^b^ Organic content from the CHNS elemental analysis, ^c^ Organic content from the thermogravimetrical analysis without taking into account hydration water.

## Supplementary Materials

The following are available online at https://www.mdpi.com/1996-1944/12/12/1953/s1, Figure S1: XRD patterns of Al-ITQ-HB hybrid materials prepared with HB as organic spacer agent: (**a**) microwave system (15 min) and (**b**) standard solvothermal synthesis conditions (24 h), Figure S2: TEM images: (**a**) Al-ITQ-EB, (**b**,**c**) Al-ITQ-HB, and (**d**) Al-ITQ-DB samples. Scale bars correspond to 20 nm for (**a**,**c**,**d**) micrographs, and 50 nm for (**b**) micrograph, Figure S3: (**a**) Argon adsorption isotherms and (**b**) Hörvath-Kawazoe pore size distribution of Al-ITQ-EB, Al-ITQ-HB and Al-ITQ-DB hybrid materials.
