# Supplementary material for: Expandable Layered Hybrid Materials Based on Individual 1D Metalorganic Nanoribbons
Source: Materials (Basel). 2019 Jun 17;12(12):1953. doi: 10.3390/ma12121953 (PMC6631333; doi:10.3390/ma12121953)
Supplement: Supplementary file 1 [file materials-12-01953-s001.pdf]

materials

MDPI

Supplementary Information

Expandable Layered Hybrid Materials Based on Individual 1D Metalorganic Nanoribbons

Jose Maria Moreno, Alexandra Velty and Urbano Diaz *

Instituto de Tecnología Química, Universitat Politècnica de València-Consejo Superior de Investigaciones Científicas, Avenida de los Naranjos s/n, E-46022 Valencia, Spain; josemmorenorodriguez@gmail.com (J.M.M.); avelty@itq.upv.es (A.V.)

* Correspondence: udiaz@itq.upv.es; Tel.: +34 96 3877800

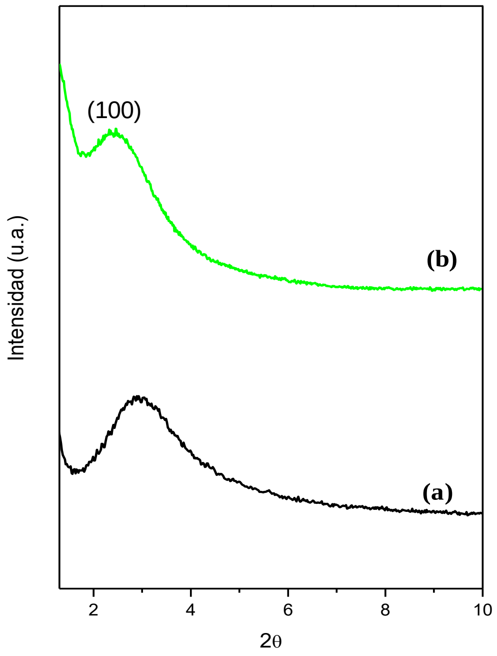

Figure 1. XRD patterns of Al-ITQ-HB hybrid materials prepared with HB as organic spacer agent: (a) microwave system (15 min) and (b) standard solvothermal synthesis conditions (24 h).

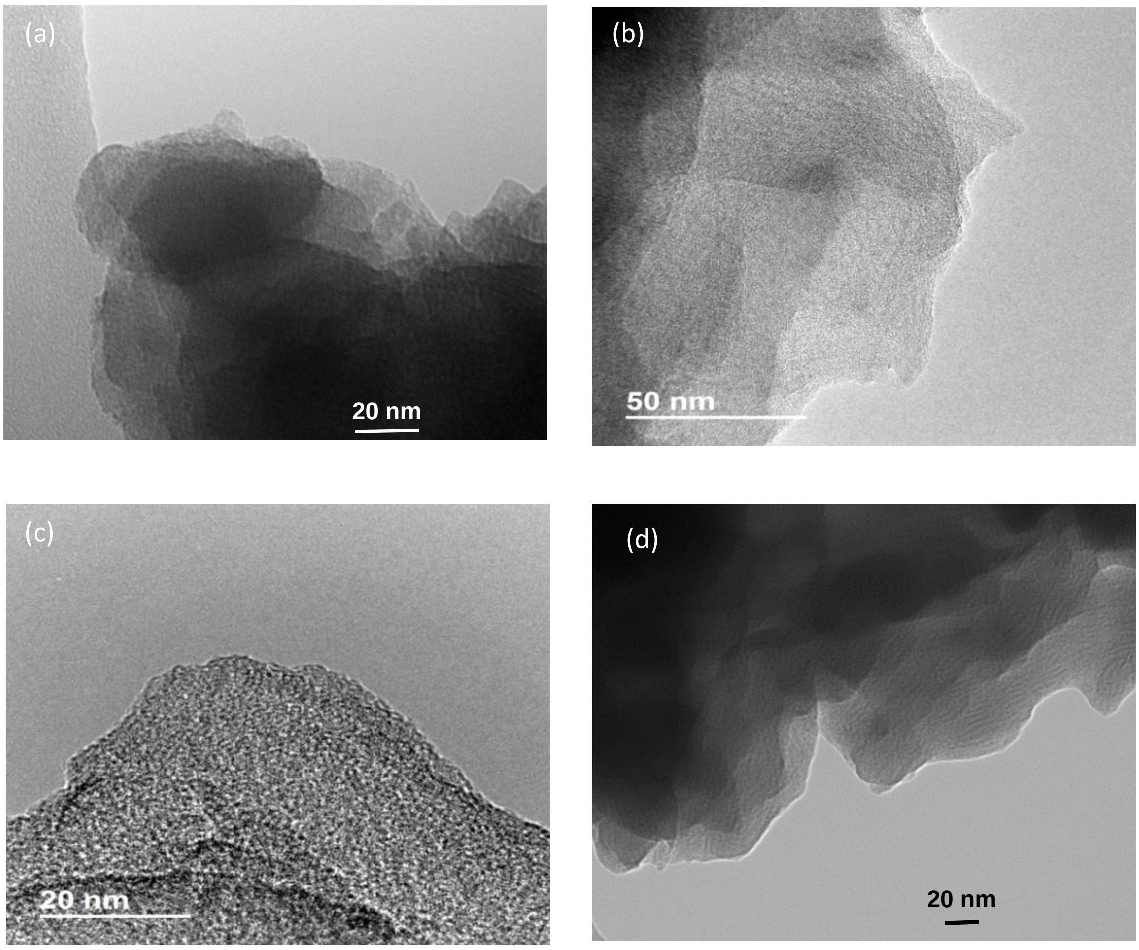

Figure 2. TEM images: (a) Al-ITQ-EB, (b) and (c) Al-ITQ-HB, and (d) Al-ITQ-DB samples. Scale bars correspond to 20 nm for (a), (c) and (d) micrographs, and 50 nm for (b) micrograph.

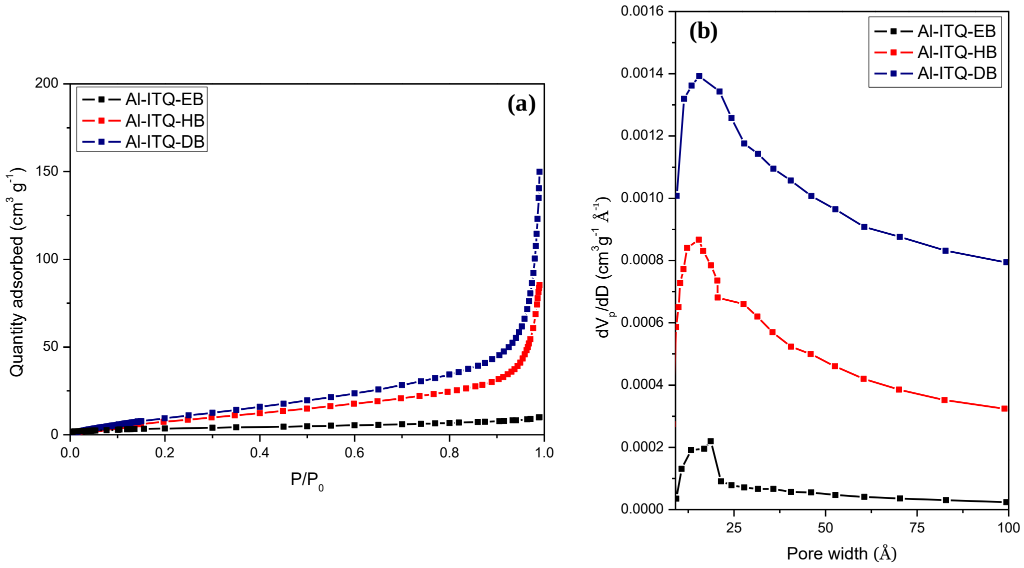

Figure 3. (a) Argon adsorption isotherms and (b) Hörvath-Kawazoe pore size distribution of Al-ITQ-EB, Al-ITQ-HB and Al-ITQ-DB hybrid materials.
